# Cancer of the penis associated with HIV: a report of three cases presenting at the CHU cocody, ivory coast

**DOI:** 10.1186/s12894-015-0101-y

**Published:** 2015-11-16

**Authors:** P. G. Konan, C. C. Vodi, A. H. Dekou, A. Fofana, E. E. Gowé, K. Manzan

**Affiliations:** Service d’Urologie, CHU de Cocody, Abidjan, Ivory Coast Africa

**Keywords:** Cancer, Penis, HIV, AIDS

## Abstract

**Background:**

We describe three cases of advanced penile cancer associated with HIV infection.

**Case presentation:**

Advanced penile cancer associated with VIH infection were discovered in three patients aged respectively 47, 56 and 40. The prognosis was extremely poor. Two patients died without receiving any treatment and one patient was lost to follow-up after refusing all treatment proposed.

**Conclusion:**

There appears to be a link between HIV infection and penile cancer with concomitant HIV infection worsening the prognosis of the disease.

## Introduction

Cancer of the penis is extremely rare. In Europe and the USA, the incidence of this form of cancer is estimated as 1.0 per 100,000 men [1]. The incidence varies between different countries worldwide with a higher incidence in Hispanic individuals, in Brazil and in Uganda [2]. Circumcision in the perinatal period or before puberty has a preventative role, but not circumcision carried out in adulthood. Early circumcision decreases the risk 3–5-fold [3], probably by improving local hygiene. Cancer of the penis is usually a disease of older men and its incidence increases with age. The peak in frequency occurs between 60 and 70 years of age [1, 2, 4]. In 95 % of cases penile tumours are squamous cell carcinomas [5]. Penile cancer may present as a flat or ulcerated exophytic papillary lesion, with the latter having a worse prognosis. The most common locations are the glans (48 % of cases) and the foreskin (25 % of cases). Penile cancer patients with advanced disease have a poor prognosis. Grades 2 and 3 disease, T3 stage and positive lymph nodes are adverse prognostic factors for cancer-specific survival in penile squamous cell carcinoma [6, 7]. In a study of eight patients with metastatic penile cancer, the longest and shortest survival times (from diagnosis of the primary cancer to death) were 16 years and 9 months, respectively [8]. Other, rarer histological forms of penile cancer such as melanoma and sarcoma may also occur [9].

There is a higher risk of penile cancer in patients with AIDS although it is not possible to conclude formally about a causal link with immunosuppression [10]. An increasing number of cases of penile cancer associated with HIV are being reported in the literature [1, 10, 11]. The aim of this report is to describe three cases of penile cancer associated with HIV that presented at the Urology Department of the CHU Cocody and to review the literature.

## Case reports

### Patient 1

A 47-year-old teacher was admitted to the CHU Cocody on the 10^th^ February 2010 with a tumour of the glans evolving over a period of 2 years. He had been circumcised when he was 17-years-old, was married and was the father of 10 children. He has also participated in unprotected extra-marital sexual intercourse. One of his partners had died a year earlier without admission to a health centre; the cause of her death was unknown. He was a drinker and a smoker. His smoking habit was 20 packets-year.

**Fig. 1:**
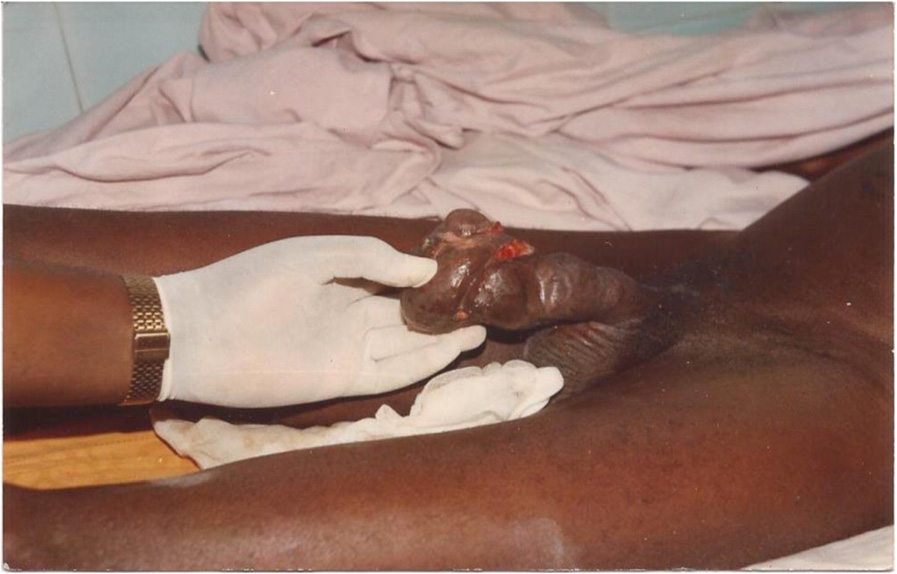
Cancer of the penis (patient 1), view 1

**Fig. 2:**
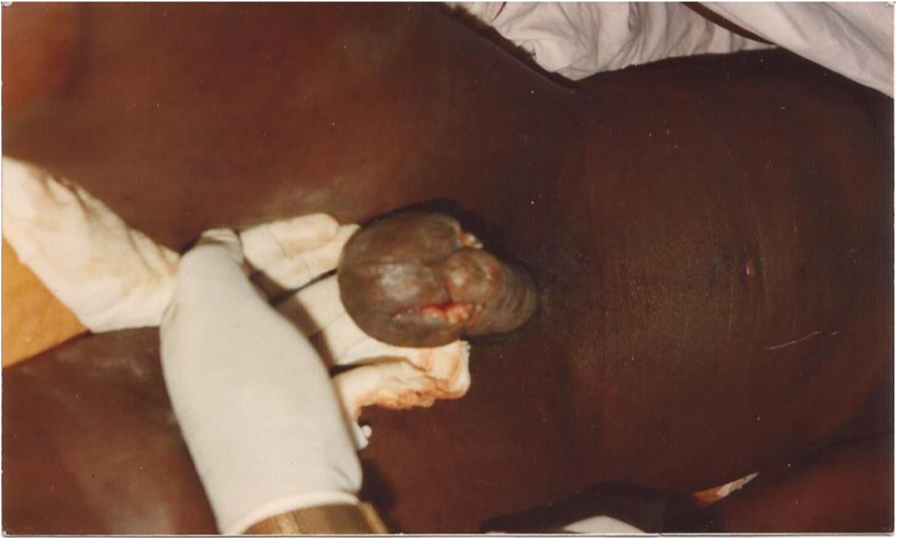
Cancer of the penis (patient 1), view 2

**Fig. 3:**
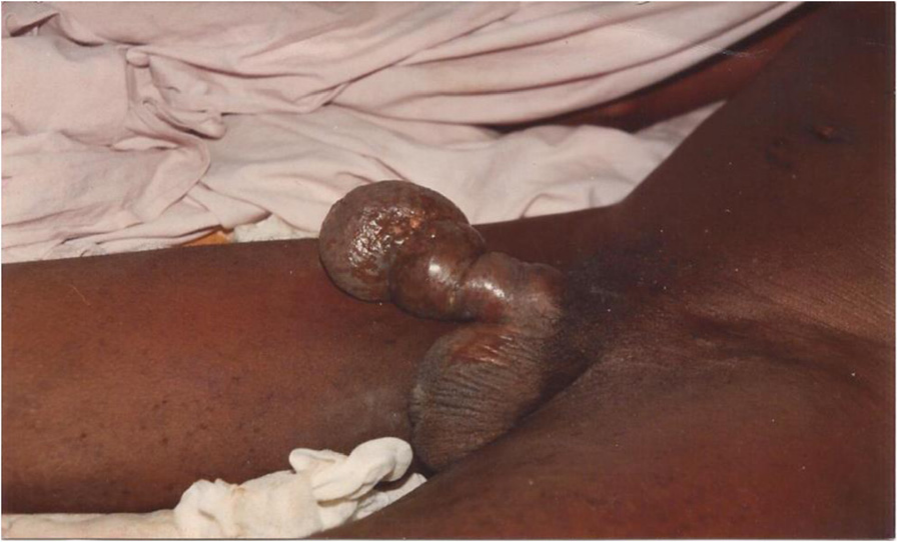
Cancer of the penis (patient 1), view 3

On physical examination, his general state was good. A hard, painless tumour was noted on his penis infiltrating the glans and approximately 3 cm of the cavernous body (Figs. 1, 2, 3). There was no superficial inguinal lymphadenopathy. Micturition was normal (Fig. 1). A diagnostic incisional biopsy of the tumour with histological examination revealed a mature and invasive differentiated squamous cell carcinoma of the penis. No metastases were discovered. The tumour was TNM stage T4N0M0. No partial or complete penectomy was performed. Serology was positive for HIV-1. The patient was lost to follow-up after the announcement of his examination results and he did not receive any treatment.

### Patient 2

**Fig. 4:**
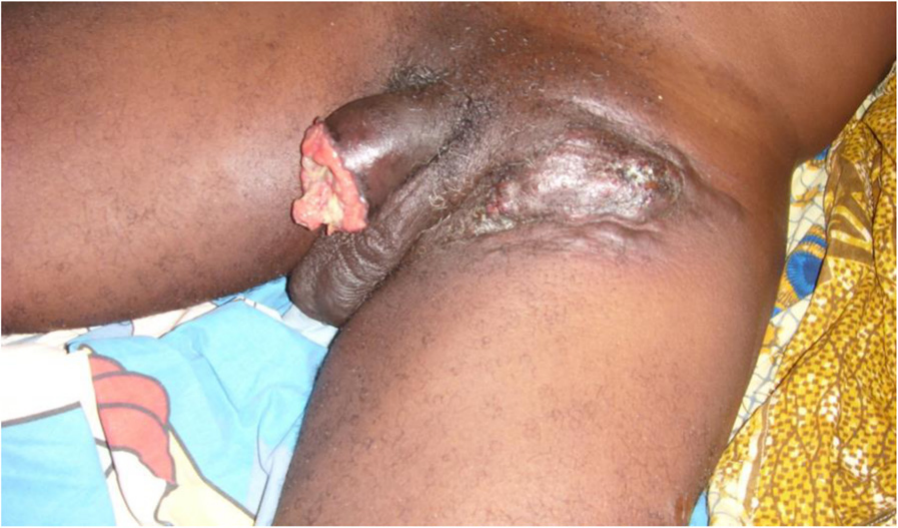
Ulcerative carcinoma with spontaneous amputation of the glans and widespread inguinal adenopathy at the point of ulceration (patient 2)

**Fig. 5:**
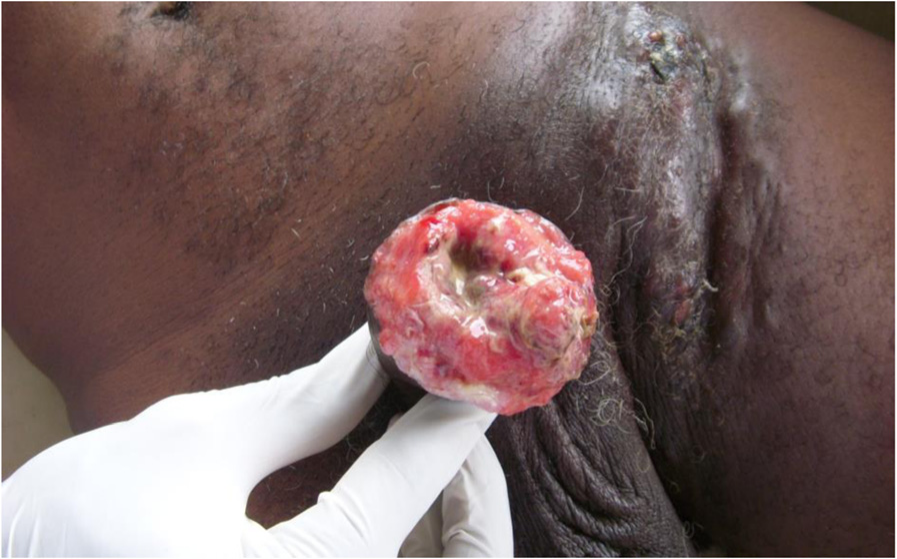
Cancer of the penis (patient 2)

**Fig. 6:**
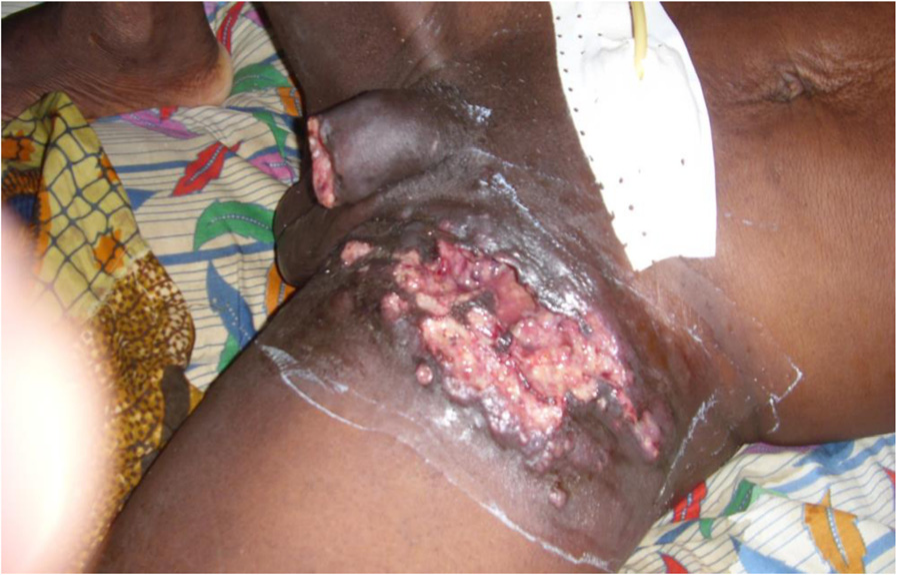
Cancer of the penis (patient 2). Ulceration of the inguinal lymph nodes; urine drained by cystostomy

A 56-year-old retired soldier was admitted to the urology emergency department of the CHU Cocody on the 12^th^ November 2010 due to a change in his general state, spontaneous amputation of the glans, left suppurative inguinal adenitis and acute urinary retention. His first wife had died 4 years earlier from cervical cancer and he had remarried. He was the father of seven children. His previous history was unremarkable. He was a non-smoker. On physical examination he was in poor general state and spontaneous amputation of the glans was noted, with an irregular crateriform wound on the penis shaft and ureteral obstruction (Figs 4, 5, 6). Polycyclic, painful and ulcerated adenitis was noted in the left inguinal area. A globus vesicalis was found. He was febrile with a temperature of 39 °C. The wound was swabbed, a sample of urine was taken and a sub-pubic cystocatheter was inserted. The patient’s condition improved with antibiotics and hydroelectrolyte rehydration. A diagnostic incisional biopsy of the lesion with histological examination revealed a mature and invasive differentiated squamous cell carcinoma of the penis. No metastases were discovered. The tumour was TNM stage T4N + M0. No partial or complete penectomy was performed. The patient was seropositive for HIV-1. He died 10 days after admission. The cause of death was unknown but was attributed to fulminant progression of the penile cancer associated with HIV/AIDS.

### Patient 3

**Fig. 7:**
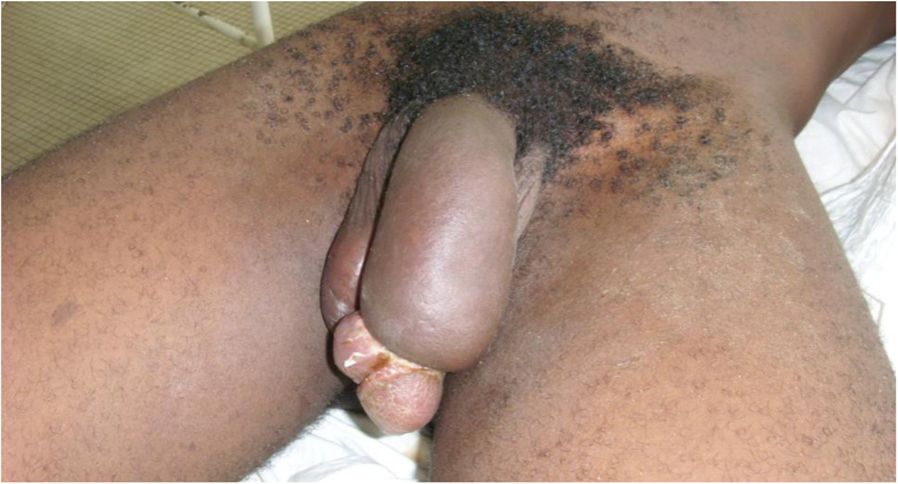
Paraphimosis seen in patient 3 on admission, view 1

**Fig. 8:**
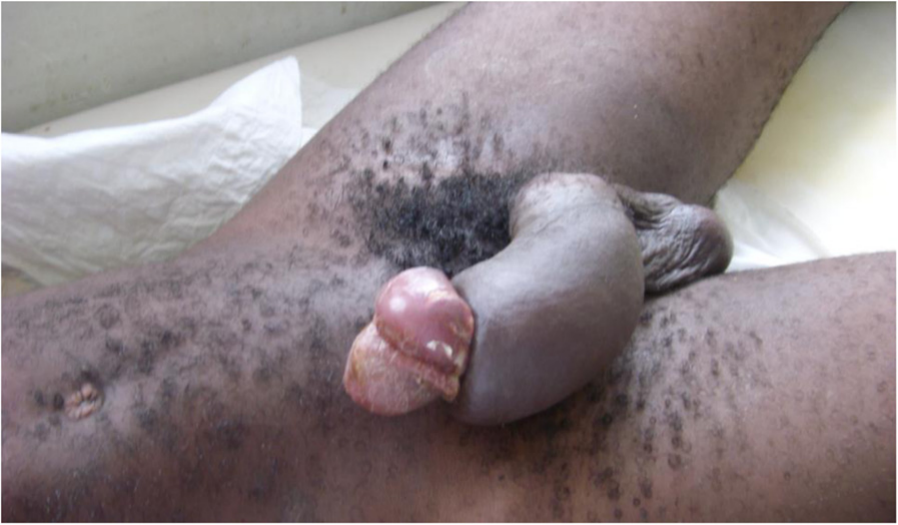
Paraphimosis seen in patient 3 on admission, view 2

**Fig. 9:**
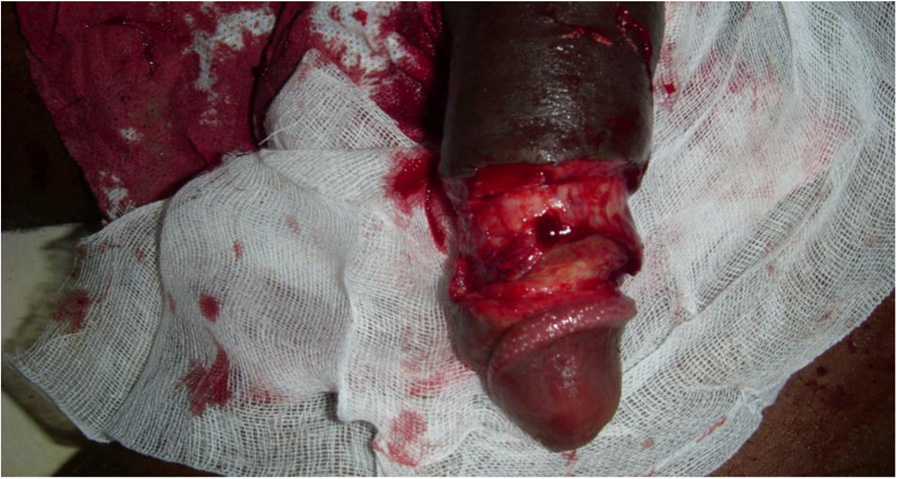
Penile tumour in patient 3 seen after circumcision and biopsy for histological examination

**Fig. 10:**
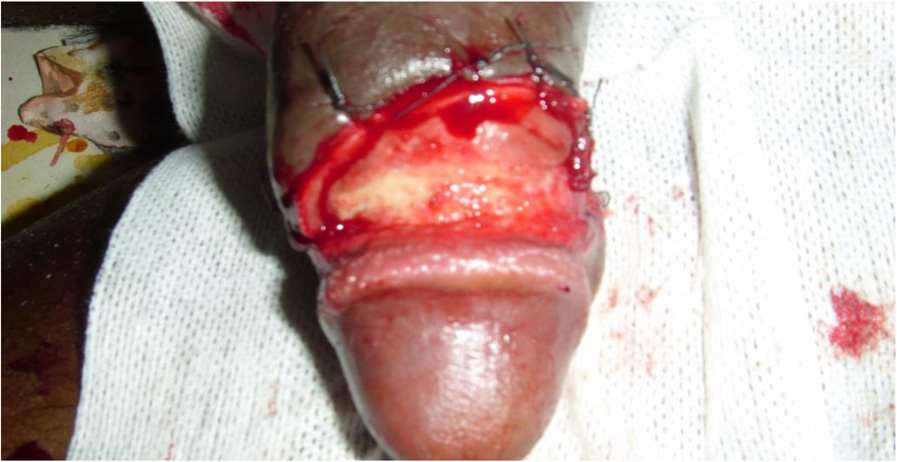
Penile tumour in patient 3, view 2

A 40-year-old statistical engineer was admitted to the urology emergency department with paraphimosis on the 6^th^ June 2011. He was not married and had no children. He had multiple partners and participated in unprotected sexual intercourse. He was not diabetic or hypertensive and did not smoke or drink alcohol. On examination, strangulation of the glans by the foreskin was noted (Figs 7 and 8). On penile palpation, pain in the glans and induration of the penis were noted. His general state was good and he was apyrexial. He had no palpable inguinal lymphadenopathy. After an emergency circumcision, a biopsy was taken of the induration of the penis (Figs 9 and 10) and histological examination revealed a squamous cell carcinoma. He was seropositive for HIV-1. No metastases were discovered. The tumour was TNM stage T3N0M0. He died 15 days later without receiving any specific treatment. The cause of death was unknown but was attributed to fulminant progression of the penile cancer associated with HIV/AIDS.

## Discussion

Cancer of the penis is extremely rare. In Europe it represents 0.4-0.6 % of cancers of men and its incidence is decreasing. In Finland, the incidence decreased from 1.4 per 100,000 between 1971 and 1975 to 1.0 per 100,000 between 1991 and 1995. In contrast, in some countries in Africa and Asia, the incidence of this disease is very high and represents 10–20 % of cancers of men [2]. In other regions of Africa, where few cases have been published or most data are old and scattered, the incidence of penile cancer appears to be low. Kebe et al. estimated the incidence of penile cancer as 2.66 % of cancers of men in the Ivory Coast in 1987 [12]. In Senegal, in 1997, the prevalence reported by Guèye et al. was less than 1 % [13]. Finally, in the Congo, only three cases have been reported, two by Bouya et al. [3] in 2000 and one case by Odzébé et al. [14] in 2010.

Cancer of the penis in the Western world is usually a disease of older men and the peak incidence of infiltrating lesions is found around 80 years of age [2, 15, 16]. In Africa, it occurs in younger subjects, aged <50 years. This was the case in our patients and in the reports of other African authors [1, 3, 14]. The mean age of our patients was 47.7 years. Although our cases are devastating examples of presentations of penile cancer, it has been reported that penile cancer can progress explosively in the setting of AIDS [11, 17].

The two most common locations of penile cancer are the glans (48 % of cases) and the foreskin (25 % of cases). The prognosis of squamous cell carcinoma depends on the depth of infiltration, its histological grade and the existence of blood, lymphatic or peripheral nervous system metastases [4, 6–8]. The main risk factors (Odds ratio >10) are maceration and a lack of local hygiene as a result of phimosis [4]. This was the case with our third patient whose cancer was discovered during paraphimosis. Other risk factors including chronic inflammation (balanoposthitis, sclera-antrophic lichen), photochemotherapy with psoralen and ultraviolet A, sexual habits (multiple partners, early first sexual relationship) and a previous history of condylomas are associated with a 3–5-fold increase in risk of penile cancer [4]. All of these sexual risk factors were found in our patients.

Squamous cell carcinoma (95 %) occurs in two main forms with a different evolution and prognosis: exophytic papillary lesions which have a late and rare lymph node spread and ulcero-infiltrating lesions which are rapidly associated with lymph node invasion and have a poorer prognosis [18, 19]. Two other forms of cancer merit particular mention: basaloid squamous cell carcinoma affects younger men, is more aggressive and is often linked to human papilloma virus (HPV) and verrucous carcinoma, which is a locally malignant lesion only.

Prior infection with HPV (particularly HPV-16) has been recognised as a risk factor for penile cancer [19–21] and for lesions with a higher histological grade [20–22]. For some authors, infection with HIV is also considered as a risk factor for penile cancer [1]. Smoking is also a risk factor for penile cancer although two of our patients had never smoked. HIV is an aggravating factor for penile cancer and accelerates the progression of the disease. This was the case for two of our patients. The last patient died before the results of the biopsy. The second patient was lost to follow-up at a terminal stage of the disease with acute evolution of the lymph nodes towards ulceration and a rapidly fatal outcome.

The pathogenesis of malignant tumours linked to HIV infection is poorly understood but their association with specific viruses such as Epstein-Barr virus (EBV), human herpes virus type 8 (HHV8) and some HPVs has been clearly demonstrated. HIV could also play a direct role in tumourigenesis [5]. During the HIV epidemic it quickly became apparent that the risk of developing a malignant tumour is higher in subjects infected with HIV than in the general population [5]. Kaposi’s sarcoma, early malignant non-Hodgkin’s lymphoma (MNHL) of the central nervous system, systemic MNHL and invasive cancer of the cervix are events that classify AIDS in the CDC classification [5].

In industrialised countries, patients infected with HIV have a 2-fold higher risk of presenting with a malignant tumour (other than Kaposi’s sarcoma or MNHL) than the general population of the same age and sex [5]. The risk of lung cancer is 2-fold higher in HIV infected individuals, probably due to higher tobacco consumption in this population [5]. A study carried out in the USA distinguished, among the cancers occurring in HIV-infected subjects, those linked with immunosuppression and those occurring at higher rates in this population due to other factors [5]. In addition to lung cancer, penile cancer, because of frequent HPV infection, and malignant tumours of soft tissue, due to probable errors in the differential diagnosis with Kaposi’s sarcoma, were more frequent in the HIV-infected population without any relation to the degree of immunosuppression. In contrast, cancer of the lip and testicular seminoma appear to be linked to immunosuppression. Finally, skin cancers (other than melanoma) are more frequent during HIV infection [5]. However, the incidence of other cancers that are common in the general population, such as breast, prostate or colon cancer, is not increased in patients with immunosuppression. Furthermore, as can be observed in patients who have undergone organ transplantation, the improvement in immune status obtained by decreasing the intensity of immunosuppressive treatment may sometimes allow complete regression of the tumour lesions. Defining the current impact of HIV infection on invasive bowel cancer and anal cancer has important implications on an individual and collective level, as screening programmes for anal and genital tumours currently exist.

With the improvements in survival brought about by current and new antiretroviral treatments, it is probable that the risk of developing or dying from cancer during HIV infection will increase in the future [5]. The impact of antiretroviral treatments varies according to the type of tumour. After immune restoration, patients are no longer at risk of developing Kaposi’s sarcoma. Conversely, patients with a previous history of severe immunosuppression remain at risk of developing MNHL. Unfortunately, adapted management of our patients was not possible due to the late discovery of their disease.

In conclusion, there appears to be a link between HIV and penile cancer with concomitant HIV infection worsening the prognosis of the disease. There is a benefit in systematically screening HIV-positive patients for penile cancer in order to discover tumours in the early stages which can be treated conservatively. Conversely, the discovery of cancer of the penis should systematically lead to screening for HIV.

## Consent

Informed consent was obtained from patient for the publication of this report and any accompanying images.
